# A youth-informed approach to mental health service websites: ‘so people can actually connect’

**DOI:** 10.1093/heapro/daaf068

**Published:** 2025-05-22

**Authors:** Senuri Panditharatne, Kerry Gibson

**Affiliations:** School of Psychology, Waipapa Taumata Rau University of Auckland, Tāmaki Makaurau Auckland, 1010 Aotearoa, New Zealand; School of Psychology, Waipapa Taumata Rau University of Auckland, Tāmaki Makaurau, Auckland, 1010, Aotearoa, New Zealand

**Keywords:** youth, help-seeking, mental health, mental health services, online communication, website design

## Abstract

There are well-recognized barriers that prevent young people from accessing timely mental health support and there is an opportunity to promote their engagement with professional support through websites associated with a mental health service. This Aotearoa, New Zealand-based study aimed to identify the elements of a website that young people believed would improve engagement with the service. A co-design method enlisted young people’s expertise in making recommendations for mental health service websites. Sixty-seven young people, aged 16-24 years, took part in one of six participatory workshops. The data was analysed using reflexive thematic analysis. Recommendations were for the inclusion of information that validated young people’s help-seeking; showed them exactly what it would be like to use the service; helped them to make informed choices about the support they wanted, and told them what steps they needed to take to get access to the service. Recommendations also included the use of personal stories from other young people illustrating the value of using the service, and for information to be conveyed in a tone that was authentic, respectful, and nonjudgemental. Young people also wanted good functionality from a website and preferred video and other visual modes of presentation. This research offers clear recommendations for websites aimed at improving youth engagement with professional mental health support. A youth-informed approach to website design has the potential to overcome some of the barriers that prevent this population from reaching out for help.

Contribution to health promotion

- Overcoming the barriers that prevent young people from accessing timely professional support is essential to addressing rising rates of youth mental health problems.
- Mental health service websites can be a vital tool for promoting better youth engagement with professional support.
- Young people’s own understanding of mental health and familiarity with youth online practices means that they can recommend strategies for developing more engaging mental health service websites.
- To better reach young people, mental health service websites should challenge stigma, provide clear information, preferably in video form, offer relatable accounts of service use, and offer a high level of technical functionality.

## Background

Although young people have one of the highest rates of mental health problems, few of those who need professional support will reach out to a mental health service (Rickwood *et al*. 2005, Mariu *et al*. 2012, Kauer *et al*. 2014). Various factors have been identified as barriers to young people’s use of professional mental health support including lack of knowledge about services, difficulty in recognizing a problem as serious enough to require professional help, stigma surrounding mental health, personal investment in autonomy, concerns about confidentiality, as well as economic and geographical obstacles to access (Rickwood *et al*. 2005, Gulliver *et al*. 2010, Radez 2021). Research also suggests that those in most need of help might experience help negation during periods of intense distress, a process that interferes with their ability to easily recognize or respond to the support available (Wilson & Deane 2010). It is therefore vital for mental health services to find ways to communicate more visibly and effectively with young people who need their help.

Young people are high internet users (Pacheco & Melhuish 2018, United States Surgeon General 2021) and increasingly look online for mental health support (Lupton 2021). While young people often prefer informal mental health resources, their explorations are also likely to bring them within reach of adverts and websites for professional mental health services (Pretorius *et al*. 2019). In this digital age organizations need to have an online presence that increases, not only the visibility but also the credibility of their services.

There is an emerging body of research evaluating mental health websites that point to features that potential service users value; these include clear information about mental health and the services offered, easy navigability, as well as implicit messages about the trustworthiness of the service (Tlach 2016, Jeong *et al*. 2019). However, there are likely to be more challenges in developing websites that will be effective in engaging young people with mental health services. Some research suggests that even in cases where young people have looked at mental health service websites, this does not necessarily increase their likelihood of seeking recommended professional help (Kauer *et al*. 2014, Sanci *et al*. 2019). This suggests that youth mental health service websites are not communicating effectively to promote young people’s engagement with the support they offer.

The limited literature on young people’s preferences for mental health websites concludes that, like adults, they value the accessibility, clarity, and reliability of the information they find on these websites (Kauer *et al*. 2014, Pretorius *et al*. 2019). However, one recent study that involved young people in the co-design of a wellbeing website for schools noted participants’ preoccupation with the barriers that prevented their engagement with mental health services (Grant *et al*. 2020). Young people in this study voiced the need for reassurance that their help-seeking was appropriate and that they would not face negative judgements from professionals. Participants also conveyed their familiarity with the pitfalls of online engagement, expressing scepticism of the corporate interests often reflected in the content. It was vital for this group of youth participants that online messages about mental health were experienced as authentic and genuinely caring.

It is also important to recognize that young people frequently explore health and mental health-related issues online and have a vast number of formal and informal resources to draw on in this space (Ito *et al*. 2009, Lupton 2021). With the wide array of resources available to them, young people are not simply passive recipients of information about mental health but are active and discerning users of ideas and resources that they see as meeting their needs (Gibson *et al*. 2024). It is particularly difficult for adults to keep up with the rapidly changing patterns and preferences of online usage amongst young people, and the new developments in social media platforms and technologies that drive these (Vogels *et al*. 2022). One of the most obvious solutions to this problem is to ask young people for their ideas about the design of online web material (Franck & Noble 2007).

Historically, young people have not been consulted on issues related to their own mental health. This is partly a product of representations of young people as irrational and in need of adult guidance (Kelly 2000). However, researchers are increasingly recognizing the importance of having young people actively involved in addressing issues of importance in their own lives (Prebeg *et al*. 2023). Recently, attention has been drawn to the potential to work with young people to develop health communication campaigns and resources (Thorn *et al*. 2020, Linda, *et al*. 2021). This is in line with our previous work which points to the importance of developing a youth-informed approach to developing mental health services more broadly (Gibson 2021).

Co-design research methods have been recommended to ensure young people’s participation in the development of more appropriate mental health services and resources that are a better fit for this generation of youth . While there are different strategies for conducting co-design research, some common features of this approach include a commitment to addressing the power imbalances that affect young people’s ability to share their views effectively; using creative and versatile strategies that facilitate their ability to communicate in ways that feel comfortable to them; and an iterative design that allows for feedback on ideas as they emerge (Mulvale *et al*. 2016, Thabrew *et al*. 2018). Some co-design approaches ask young people to comment on preexisting interventions or models, but in an area where there is still much to be learned, it would be preferable to ensure ideas are generated first by young people before they are harnessed into shape by professional interests.

## Methods

### Research design

This research used a co-design method to address the question: What do young people think mental health services should include in their online websites in order to improve youth engagement with their services?

The research was conducted in two phases. The details and findings of the first phase have been published in a previous article (Adeane & Gibson 2023). This first phase of the research used an innovative method of collecting interviews via WhatsApp messenger service (Gibson 2022). Semi-structured interviews were conducted with 37 young people aged 16-23 years to ask them what kind of online messages were more likely to prompt young people to seek professional mental health support. This research identified three main criteria for effective messages: those containing clear and detailed information about the mental health services available to young people, information that challenged popular misconceptions about mental health services, and those that contained information that young people could relate to.

The current article captures the findings of Phase Two of the co-design process. This consisted of workshops with young people aimed at evaluating the suggestions that had emerged from Phase One and translating these into practical recommendations that could be used to guide youth mental health services on how to design effective websites.

Our research method drew from suggestions for carrying out co-design projects with young people (Thabrew *et al*. 2018). Recommended strategies include an iterative process starting with the lived experiences of end users before introducing professional knowledge. They suggest moving from broader ‘blue sky’ thinking towards more practical recommendations, reflected in the shift in focus from Phase One to Phase Two of our research. Our research was also strongly influenced by a youth empowerment perspective that prioritizes young people’s voices on matters of concern to them (Wyn & Harris 2004).

This research received ethics approval from the University of Auckland Human Participants Ethics Committee (reference number 023698). All participants gave written informed consent for participation in the study. The study was carried out in Aotearoa, New Zealand (NZ).

### Participants

Participants from Phase One of the project were invited to participate in Phase Two, but we had a limited response to this invitation, with only six participants from the first study making themselves available. We re-advertised participation to youth groups and schools to extend the sample. Our advert asked for participants between the ages of 16 and 24 years who had ‘an interest in youth mental health’ and were regular users of the Internet. They were not required to have personal experience with mental health issues themselves, as we hoped to capture a range of experiences, including those who were more cautious about engaging with professional mental health services. However, it became clear during the workshops that most of the participants had experienced mental health distress and had explored the internet for information and support for mental health issues.

The final sample consisted of 67 participants. Participants were aged 16-24 years, with all but two participants under 20 years. Fifty-nine participants were at a secondary school, five were at university, one had recently completed university and three did not provide this information. Forty-eight (72%) of participants identified as female and 19 (28%) as male. There were 47 participants that identified as heterosexual, six as bisexual, three as ‘questioning’, one as pansexual, one as queer, one as asexual. Seven participants chose not to disclose their sexuality. The participants also self-described their own ethnicity, with 36 participants identifying as New Zealand European, 13 as Asian (with origins in different Asian countries), six as European, five as Māori, two as Middle Eastern, two as Pacific peoples, two as South African European, and one as African. Most participants only provided one ethnicity but where more than one was provided, we followed the NZ conventions of prioritized identity (e.g. listing a participant as Māori where they also acknowledged NZ European).

### Data gathering

Participants took part in a workshop, each lasting approximately 90 minutes. As our priority was to ensure maximum participation from young people, and data collection occurred during a period that was disrupted by COVID-19 lockdowns (June to November 2021), we adopted a flexible approach that allowed participants to take part in either an in-person workshop or an online Zoom video workshop. Three in-person workshop groups were held in a single school and had between 16-24 participants in each of the three groups. There were three online Zoom workshops which were smaller, with between two and five participants in each.

All workshops followed the same basic structure and were facilitated by young researchers (including the first author of this article) in order to provide a less intimidating setting for young people to contribute their ideas. The in-person workshops had two group facilitators each and the online workshops had only one facilitator. The facilitators gave participants a brief introduction to the themes that had emerged in Phase One of the project. Young people were asked to discuss the themes that had been generated in the previous phase of the research and to revise these where appropriate. They were then asked to translate these ideas into practical suggestions that could be included in a mental health service website. They were given several prompt questions to help them with this task including: ‘What would your main message be to young people?’ ‘How should the message be delivered, e.g., by a person, text, a video?’ and ‘How should the message be presented so that young people will attend to it?’ Participants were then divided into break-away groups in the larger workshops (or remained in one group for the smaller ones) to brainstorm how the ideas could be translated into practical ideas for a mental health service website. Once this process was completed, participants presented their feedback to the large group and further discussion was facilitated. Participants in the in-person workshops were provided with white paper and pens to brainstorm and record their ideas. This was not possible or considered essential in the smaller online workshops, but participants were encouraged to use the chat function if they felt uncomfortable with talking.

We were conscious of the sensitivity of the subject and had in place appropriate protocols to deal with any participants experiencing distress. Workshop facilitators had some clinical psychology training and were instructed to remain alert to signs of distress amongst participants and offer support if necessary. If this on-site support was not sufficient, the facilitators were to contact the senior researcher, an experienced clinical psychologist, who could arrange a suitable referral to a mental health service. No referrals were required during the course of the research.

### Data analysis

Break-away group discussions were not recorded but all other discussion was recorded and transcribed. Notes and chat messages from the breakaway groups and large group discussions were also written down and included in the data for analysis. The data were analysed using reflexive thematic analysis (Braun & Clarke 2006). This approach was consistent with the tenets of co-design, emphasizing the importance of the researchers’ reflexive awareness of their role in the research process (Braun & Clarke 2019). This method called on us to reflect on how we, as adult researchers and mental health professionals, might understand young people’s mental health needs differently from the way they did and drew attention to how power operates in meaning-making.

In line with Braun and Clarke’s (2006) recommendations, we immersed ourselves in the data, developed codes that captured the meanings conveyed, and then crystallized these into themes. In keeping with the aim of the research, we styled these themes as ‘recommendations’ for the design of websites. While conducting the analysis, the two authors of this study, one a young student researcher training in clinical psychology and the other an older senior researcher and clinical psychologist, occupied different positions in relation to youth. This facilitated different perspectives on the data. We discussed and resolved any differences through consensual decision-making which is considered to increase the rigour of qualitative analysis (Hill, 2014).

## Results

The findings present themes reflecting participants’ recommendations for the design of mental health service websites for young people. The themes were divided into two categories; the first capturing recommendations for the information young people need to facilitate their help-seeking; and the second, suggestions for the format in which this information should be delivered to young people.

### Information to promote help-seeking

#### Normalize help-seeking: ‘It’s okay to see someone’

Participants recommended including information on websites that destigmatized getting professional support from a mental health service. They said that despite there being a greater awareness of mental health problems in youth networks, engaging with a mental health professional still carried considerable stigma amongst young people. They explained that while young people were often reasonably comfortable talking to each other about the possibility of having a mental health problem, this was different from receiving a judgement from a professional that might suggest something ‘abnormal’ about them. Workshop discussion captured the paradox that while talking about mental health was ‘trendy’, there was a long way to go before seeking help from a professional was seen this way.

Participants suggested that mental health service websites convey messages that actively challenged this view by ‘normalizing’ mental health problems. They suggested that one way of doing this was to include ‘like some statistics, maybe like, so many people experienced mental health issues. So it’s like normal’. Given that young people struggled to translate their awareness of mental health distress into help-seeking it was important to emphasise this as a reasonable step to take in the face of mental health distress. Participants elaborated that websites should deliver a strong message that it was an appropriate action when experiencing distress and that it was ‘okay to seek help’ They also suggested that websites emphasize the difference between self-reflection on mental health, to actually working with a professional to resolve these: ‘Just try to emphasize the importance of actually talking to someone else about issues’.

Participants recognized that it was harder for some groups of young people to seek help than others. They suggested that websites should contain information directly targeted to these groups, like young men, who were particularly unlikely to reach out for help. They explained that this was because men have been told that they should ‘bottle in their emotions and then sort of keep everything to themselves’. One young man, for example, said it was important for mental health services’ websites to acknowledge and directly challenge the stigma associated with men’s help-seeking: ‘So by portraying a message that says like, “Go see someone!”. Normalize the struggle, you know? And by making a bunch of posts saying like, it’s okay to talk to someone face to face’.

Participants explained that young people also often felt that their problems were not sufficiently serious to require professional advice or that they did not ‘deserve’ this help. They emphasized the importance of websites providing reassurance to young people that their problems were ‘valid’ and that they ‘deserved to get better’. They suggested including resources that would help young people realize their problems warranted help including, for example, online mental health ‘self-quizzes’ to help young people ‘diagnose’ their mental health problems and to help them realize that professional help was needed. As one young person explained: ‘So like, for example, if you get nine out of 10 of these things, you’re depressed. So these can be helpful for like guiding us’.

#### Explain how therapy works: ‘Just to show what it’s like’

Participants recommended that websites include clearer and more detailed information about how therapy and other interventions actually worked. They discussed that young people’s knowledge of mental health services was often based on representations in popular media (e.g. television and film) and that ‘a lot of people don’t know what it’s like in person’. One participant reflected on how they had been deterred from seeking help because they relied on outdated ideas about how mental health therapies worked:

I mean, I had a certain idea, and I didn’t realize, you know, that that barely happens these days… one thing that would be stopping me would be because I don’t know what it’s going to be like.

Participants suggested various strategies to help demystify what happened in a session with a mental health professional. For example, they suggested that service websites include videos showing ‘what actually happens inside’ and that ‘watching a mock counselling session’ could help young people see what it was like to get help. One participant suggested making brief videos on the website that young people could watch: ‘Like a little, clip of like, a fake therapy session. Just not like, not like an hour long one, like a 30 second one you know. Just to show what it’s like’.

Participants also recommended that services should consider other ways for young people to find out more about how their services worked. One participant, for example, suggested services might create an interactive website with input from practitioners and service users: ‘Like a website where you can communicate with people like either actual therapists or like people who have been to therapy. And you can, like, ask questions about what it’s actually like in there’.

Participants also felt it was important to give young people a realistic understanding of the commitment therapy involved. They felt it was important for young people to realize that professional help was not a ‘silver bullet’ and that they should be prepared to persevere to get the outcome they wanted. One participant, for example, spoke about how young people should made aware of how long it takes to address mental health problems effectively: ‘So like, it’s not instant fix, one session isn’t gonna, like change your life forever, I don’t know. And it is worth it. If it does take time, even like being better, you know, it’s good’. Participants explained that these realistic messages about mental health services would prevent young people from becoming disappointed at a lack of an immediate result and help quash some of the disinformation in their networks that suggested therapy was ineffective.

#### Tell them exactly how to get help: ‘Information about where you can go’

Participants recommended that websites outline a clear process for gaining access to a service, information that would clarify whether a young person met the criteria to use the service, and what steps they needed to take to get the help they wanted. They explained that young people were often frustrated with websites that instructed them to ‘get help’ without telling them exactly *how* to get help. They wanted the process to be outlined in step-by-step instructions, broken down ‘into little chunks’. As one participant put it: ‘Like, it’s no help just being like, “you need help”. Like, where do I actually go to do that? And like, what is the expense gonna be? How do I get there?’ They recommended that mental health services should pull all this information together in one place on their websites so that young people could easily find it. As one participant elaborated: ‘Use the platform, sort of as like a first point of contact that can get used as a guide and information about where you can go and how to access the help you need’.

Participants went on to suggest that services might also simplify the help-seeking process on these websites by offering young people self-assessment tools to point them towards the professional or service that might be most appropriate for them: ‘like self-assessments and stuff, and just information about where you can go to next and who you can get help from and what sort of experience they have and things like that’.

Participants noted that the variety of different professionals working in mental health did (e.g. a psychiatrist, a psychologist, a counsellor) led to young people feeling confused about what exactly each profession did, what differences there were between them, and which were appropriate for them and their situation. They said that they would find it helpful if mental health websites clearly explained the differences between the professions: ‘Like, ‘cause obviously not everyone knows what like a counsellor is and like, what a psychologist is… so maybe just getting a better idea on what they all are’.

Practical considerations were also seen as important in determining the fit of the service for the young person. Participants noted that websites often advertised a service without making it clear whether and how young people could access it. As one participant emphasized, it was important to know: ‘Whether it’s like, you can access them from anywhere in Aotearoa New Zealand, over the phone, or Zoom or if that person’s available in your city for face to face, or if they don’t do face to face at all’.

Participants often referred to the cost of therapy being a barrier to young people seeking professional help. They recommended that websites provide information that would help them identify whether they could afford to get help from a service. One participant suggested websites use ‘pop up quizzes’ so ‘they can say, you know, like, that’s, like designed for your city that shows like local therapy, therapy, like areas and like, therapists and like affordable ones. And like free ones’.

Many participants also recommended making details of each practitioner available online so that they could make an informed decision on which services were compatible with their personal preferences and identity needs. One participant explained that she would be much more likely to respond to a service where the website had a full description of each counsellor or therapist and information about their therapeutic style and history:

The ones [the websites] that sort of just had a list of names or a face, I was less likely to go oh yeah I want to see that person whereas the sites that had a full breakdown, photos of each person, a blurb about the sort of like what they’d worked, who they’d worked with in the past what their specialties were, I was more likely go okay, yeah, that person there is really suitable for me.

Similarly, another group of participants suggested having practitioners post a video alongside their written information so a young person could get a better sense of the practitioner as a person. One participant explained why this was important to keep young people engaged with the service:

Obviously, you don’t click with everyone that you meet. And so if there’s like short little videos, then you can like pick the best one which you think you’d like click with.

### Format for delivery of information

#### Use relatable stories: ‘More likely to listen to a 16-year-old’

Participants recommended that website information be conveyed in ‘story’ form by people they could relate to, rather than just communicated as a set of facts. They explained that young people would be most likely to respond to personal accounts from people who seemed similar and had experienced the same struggles as them. As one participant put it: ‘We thought, it’s really helpful to include personal stories, so people can actually connect to it instead of just like, throwing information out there’. Another participant explained that young people would be more inclined to reach out to the service for help if they could hear from other young people who had been through this process: ‘Like just talking through, like, what helped them. What didn’t help them. What steps they took. So if someone can relate to that, then they can maybe take those similar steps’.

Several participants suggested that mental health services consider using social media influencers to convey information on their websites. One participant reasoned that this is because young people often looked up to, and trusted, social media influencers more than other sources: ‘You, as a young person, you’re more likely to listen to your idols and people you follow. The people you like, as compared to just some random ads that come up across your feed’. One participant explained that this strategy would help young people feel that they were not being coerced by adults to do something against their will: ‘Like your parents tell it in an enforcing manner. When celebrities tell it’s gonna be an encouraging manner. So that would be a better approach, and rather than pushing them, they get pulled in’.

However, rather than services utilizing older, high-profile influencers to deliver their messages, participants felt that services should rather enlist the help of ‘soft influencers’ who were closer in age and led similar lives to them. One participant explained: ‘Like, I think I’m more likely to listen to a 16-year-old who has a similar life to me than, I don’t know, like a 30-year-old celebrity’. Another participant had the idea of using a local youth celebrity to deliver these online messages in video format and provided this example of what he might say:

…he just has a little video that says, like, hey, guys, like, it’s okay to get help. I’m here. And it’s okay. I have been through that, or whatever. I’m just like, normalizing it, and showing that it’s okay. And this is how you go to it. This is what to do. It’s completely okay. It’s completely normal.

Other participants agreed, adding that showcasing stories of mental health recovery from people from various backgrounds was crucial. One participant explained that this would mean young people could find someone they could personally relate to:

‘Cause I think everybody’s so different. So I think people need to just find someone that they can relate to. Or that’s like them, or like talks like them, or speaks like them, looks like them. So then they can form that emotional connection to then act on it in their life.

#### Getting the right tone: ‘They are trying to help you’

Participants explained that young people were very sensitive to the tone of the messages they received and recommended that websites communicate a ‘genuine’ commitment to helping young people. They expressed particular scepticism about content with a commercial interest at stake. As one participant explained: ‘Yeah, making sure it doesn’t come across as like, the only reason they’re doing this is because they’re being paid a lot. Yeah. Like you want genuine’. They explained that youth were attuned to recognising when people were providing endorsements for money because this was something that commonly occurred with big social media influencers:

If it’s just like an ad, you know, as they were saying, like a script. Then it’s gonna just seem you know, like a lie kind of. I’d be like, ‘Oh, okay, they’re just being paid to say that’. It’s not believable.

When online messages felt disingenuous, participants described how they would quickly disengage. A participant gave this account of their experience on the popular streaming website, YouTube, as an example:

You see a lot of people doing ad reads before videos, and they all have the same script every single time. And it’s two minutes, and you go in, and you’re like, this video was sponsored, and you’re like, skip two minutes.

Participants said that young people were likely to be more responsive to website content where the motivation was genuine concern for their wellbeing. They explained that young people would respond better to more ‘natural’ and unscripted endorsement of a service:

So if you, sort of naturally include something that you genuinely feel is worth talking about into your content, then you’ll generally have a better response to that content, rather than interrupting it halfway through to read a script, given by a company.

Participants also explained that young people were sensitive to signs of condescension in communications from adults. One participant, for example, explained why age-targeted ads which attempted to speak to ‘the youth’ were ineffective and often experienced by young people as patronizing:

I feel like a lot of the reason a lot of campaigns fail for me is that they’re targeted at the youth... I want a much more mature approach. [Young people] want to be treated like adults. They want to be treated like everybody else.

In addition, participants’ discussion suggested that young people did not take well to being told what to do. They recommended that mental health services adopt a respectful approach, recognizing that young people were thoughtful and discerning about their own mental health. One participant, for example, suggested that youth would prefer that, rather than just being given information, they were encouraged to reflect on their own mental health: ‘Like questions even. “Are you experiencing dot dot dot?” And then you kind of go, oh I am actually, I’ll go back and have a look at what that was’.

Participants also reflected awareness of young people’s concerns about negative judgements on their mental health from professionals and said young people were looking for reassurance that they would be accepted. As one young person put it: ‘Yeah, I think it [the tone] definitely has to be warm, because otherwise people aren’t really gonna gravitate towards it or listen to it …. If it’s like really judgmental, people aren’t really gonna listen to it’. Given the anxieties that young people had about being judged for their mental health problems, participants recommended that this should be one of the first messages that a website conveyed: ‘Maybe like simple quotes, like: “People should not be judged for anything”’.

#### Make it quick and responsive: ‘So you click it’

Participants emphasized that young people were accustomed to sophisticated digital technology and would be put off by slow or clumsy website operations. They discussed the importance of having quick and easy access to the information they needed:

Have the information, like really easy to find, and like all there, because we’ve got one of the shortest attention spans. And if we can’t find it, we’re not going to look for it.

Participants also recommended using video, instead of text, to convey information. They emphasized the appeal of video material to young people, noting that this was the primary content on some of the currently most popular social media platforms, such as TikTok and YouTube. Videos were seen to hold young people’s attention more easily than text and helped to convey the authenticity of messages. However, they noted that videos should be kept ‘short and snappy’ as they did not believe that any young person ‘wants to sit through a 20-minute speech with therapists’. Together with videos, participants suggested making maximum use of the diverse visual mediums available online, like infographics or interactive slide shows, which they felt would be ‘less intimidating’ for young people than reams of text.

While websites were considered an important point of contact for young people, they also suggested that adverts be used alongside these to reach into the social media networks where young people spent their time online. Participants explained that young people would not necessarily seek out a website themselves because they did not know where to look or did not recognize that they needed help. As one young person said: ‘it’s just harder to go and search for it yourself’. Therefore, ‘pop up’ adverts that appeared in young people’s existing social media feeds were seen as a valuable way of reaching young people. As one participant put it: ‘Kind of a little bit put in your face. So you’re actually kind of forced [to see it]’. However, participants elaborated that it was important that these adverts break through young people’s resistance to engaging with intrusive advertising on their social media feeds. One participant, for example, suggested ‘unskippable’ advertisements on YouTube that young people would be compelled to watch before they could view their chosen video.

Participants also said it was important to make sure these adverts were released on a range of social media platforms to reach groups of young people who frequented diverse networks. They suggested that the algorithms used by social media could help promote the message to young people who might be particularly in need of help: ‘So TikTok, like curates everything to your *For You* page. So I mean, if you have ads, that are like, based off, [the algorithm] you know, they’ll find out what’s wrong with you’. Participants explained how these ‘pop-up’ adverts should allow young people to move easily from their own social media networks through to the mental health service’s main websites to find further information: ‘So you click it and it takes you to the page, and then you click the link, and you do eventually end up at the website’.

Although young people recommended that resources could be promoted via social media feeds, they felt that the main source of information should still be located on a different platform such as a website or an app. Besides facilitating improved functionality, participants also noted that young people were very concerned about the confidentiality of their data on social media. Therefore, they suggested that young people would want, and would feel more secure engaging with, a website that was administered by a legitimate mental health organization.

## Discussion

This study offers clear recommendations for the kind of information, format, and tone required for youth mental health service websites based on young people’s own preferences.

It is recommended that youth mental health service websites should include information that challenges the stigma young people still associate with using professional support for mental health. While there has been considerable emphasis on mental health literacy for youth and efforts towards destigmatizing mental health problems (Kelly *et al*. 2007, Marinucci *et al*. 2023), there has been less attention paid to the stigma associated with using a professional service (Mitchell *et al*. 2017). There is clearly more work to be done to remove this significant barrier to young people’s help-seeking—especially for groups like young men who have to overcome personal and social barriers to do this (Lynch *et al*. 2018). Young people also need reassurance that their problems are legitimate and require professional help and that using a service could be helpful to them (Gulliver *et al*. 2010).

Young people also want to know more about what is actually involved in getting ‘help’ from a professional. Mental health services and the professionals who work for them may not realize how little the public understands what they do (McKeddie 2013, Ebsworth, & Foster 2017). Websites could make use of brief videos of therapy sessions, personal accounts from service users, and interactive Q&A sessions to demystify their services and help bridge this gap.

While young people might be misinformed by representations of therapy and mental health services in popular media (Goodwin & Behan, 2023), with their exposure to a broad array of information online, they are less likely than previous generations to simply accept that professionals know best. Young people are very aware of their own agency and right to choose (Gibson 2021). Young people want sufficient information to understand what a particular service could offer them, how their various approaches might address their issues, and how these services would meet their individual needs.

In addition, young people want to have detailed information on how to access a service. Too often, young people are told simply to ‘get help’ without enough detail on how to do this. As other research has also suggested, youth want clear information that tells them what service they might afford, whether they meet the criteria to use it, and step-by-step advice on how to go about setting up an appointment (Goodwin 2023).

Services should also consider the tone of their communications on their website and the intangible messages this delivers. Young people are deeply sceptical of commercialism and ‘fake’ messages (Hargittai 2010, Gibson & Trnka 2020). They are looking for services they can trust and that genuinely care for their wellbeing (Gibson 2021). Practical recommendations for websites include using personal accounts given by people who have experienced mental health support, delivered in a genuine and nonjudgemental tone.

The recommendations from this research reflected young people’s familiarity and sophistication in their use of digital communication technology (Ito *et al*. 2009). This generation of youth is accustomed to the efficient technologies of online gaming and social media. Unsurprisingly, they are looking for responsive websites that give them quick access to the information they seek. The video was seen as a preferred communication medium, mirroring the increased popularity of video-based social media (Vogels *et al*. 2022).

Traditionally, online material about mental health services contains abstract, static, and de-personalized information delivered through text and images. This study suggests the need for greater permeability between the offerings of the service and its online presence. Young people expect to come away from an engagement with a mental health service website with a sense of who the organization is, what its values are, and an online taste of how the engagement might play out if they were to reach out for help. This is a function of a generation accustomed to a blurring of boundaries between online and offline engagement (Boyd 2014). This is also a product of young people who remain sceptical of mental health services and uncertain about what they have to offer them (Gibson 2021).

The recommendations captured in this research would help address the psychological barriers that prevent young people from accessing appropriate and timely professional support for mental health problems (Rickwood *et al*. 2005, Gulliver *et al*. 2010, Radez 2021). This research also reiterates the importance of working together with young people when attempting to address important issues in their lives (Schelbe *et al*. 2015). In this research, we harnessed young people’s expertise in in order to improve mental health messaging directed towards this hard-to-reach population. As young people’s online preferences change, and technologies become more sophisticated, it is important that mental health promotion strategies keep up with these.

The limitations of this research include the low retention of participants from Phase One to Phase Two of the study. This might reflect the transitions common in this period of life (from school, to university, to work) that made it difficult to track youth participants over the two years during which the project was running. These recruitment difficulties were exacerbated by the many COVID-19-related lockdowns that occurred in NZ during this period. There was also limited participation from Māori and Pacific youth, which is a significant omission given the barriers to engagement with mental health services linked to racism, a history of colonization, and a poor fit between services and the needs of these communities (Fa’alogo-Lilo & Cartwright, 2021; Theodore *et al.*, 2022). While there were diverse cultural groups represented in the sample, it is perhaps telling that the data did not reflect a concern for culturally matched mental health professionals, an idea that has emerged in other local research (Mila-Schaaf & Hudson 2009, Latimer *et al*. 2022, Peiris-John *et al*. 2022). This anomaly was likely due to the method of data gathering which did not offer an opportunity for different cultural groups to discuss their views separately. This format might have restricted the extent to which specific cultural views were represented in the general discussion. In addition, the data collection methods might have excluded young people with disabilities or a lack of access to appropriate technologies. We were also conscious that while many of the participants disclosed their own issues with mental health, the study did not exclusively capture the views of those with mental health difficulties. Finally, we also recognize that the research might have reflected the challenges of a period in which youth were dealing with the intermittent challenges of COVID-19.

Despite the limitations, the strength of this study is the engagement of young people in workshops designed to challenge power and enable their own ideas. There is also extended engagement and the opportunity to build on ideas generated in an earlier stage of the project, allowing for multi-method engagement consistent with the principles of good co-design (Thabrew *et al*. 2018).

## Conclusion

The discrepancies between youth mental health rates and the number of young people accessing support indicate that a modified approach to engaging with youth is needed. It is essential that youth mental health services design their online communications with young people in ways that resonate with, and engage, young people who might benefit from their services. A youth-informed website can be a valuable way of overcoming the barriers that prevent young people from reaching out for professional mental health support.

## Data Availability

Due to ethical considerations of confidentiality the raw data used in this study cannot be made available.
